# Prediction of Sinus Rhythm Maintenance After Electrical Cardioversion Using Spectral and Vector Cardiographic ECG Analysis

**DOI:** 10.1111/anec.70105

**Published:** 2025-08-15

**Authors:** Sabri Hassouna, Marek Hozman, Dalibor Heřman, Jana Veselá, Věra Filipcová, Filip Plesinger, Zbyněk Bureš, Pavel Osmančík

**Affiliations:** ^1^ Cardiocenter, Third Faculty of Medicine Charles University and University Hospital Kralovske Vinohrady Prague Czech Republic; ^2^ Institute of Scientific Instruments of the Czech Academy of Sciences Brno Czech Republic; ^3^ Czech Institute of Informatics, Robotics and Cybernetics, Prague, Czech Republic, Department of Otorhinolaryngology, Third Faculty of Medicine Charles University and University Hospital Kralovske Vinohrady Prague Czech Republic

**Keywords:** atrial fibrillation, electrical cardioversion, prediction of sinus rhythm, spectral and vectorcardiographic analysis

## Abstract

**Introduction:**

Electrical cardioversion (ECV) remains a treatment option for atrial fibrillation (AF). The study aimed to find predictors of SR maintenance after ECV using spectral and vector cardiographic (VCG) analysis of ECGs.

**Methods:**

Consecutive patients with AF referred for elective ECV were prospectively enrolled. A digital ECG recording was obtained before the ECV and was analyzed using spectral and VCG analysis. AF activity was analyzed using spectral analysis to determine the dominant frequency (DF), RI (regularity index), and OI (organizational index). QRS complexes were analyzed using vectorcardiography to determine the dXmean, dYmean, and dZmean (derivation of VCG signals). We used Lasso Logistic Regression (LLR) in five‐fold cross‐validation for feature selection and to build combined predictive models of SR maintenance. For model training and evaluation, data were split in a 60%–40% ratio for training and testing, respectively.

**Results:**

A total of 80 patients were enrolled (age 70.2 ± 10.6 years, 49 (61%) were men, BMI 29.7 kg/m^2^). At the 3‐month follow‐up, AF recurrence was present in 36 patients (45%). The best single VCG parameter to predict SR maintenance was dZMean (OR 0.18, 95% CI 0.06–0.51, *p* < 0.001). VCG‐domain parameters combined into the LLR model showed an area under the curve (AUC) of 0.78. From the spectral analysis domain, the best predictor was DF (OR 3.54, 95% CI 1.28–10.25), *p* = 0.006; spectral features led to an AUC of 0.76 when combined in the LLR model. Clinical features did not form a model since no features passed feature selection. Combining VCG and spectral analysis features led to an LLR model with an AUC of 0.79.

**Conclusion:**

The combination of spectral analysis of AF activity and VCG analysis of ventricular activity provided more accurate predictive information than either analysis alone.

## Graphical abstract

The combination of spectral analysis of AF activity and VCG analysis of ventricular activity provided more accurate predictive information than either analysis alone. Since our ECG parameters are easy to analyze, in the future, it could become a standard part of ECG Holter recording to help in the decision‐making process when the patient is referred to cardioversion.

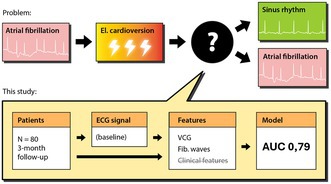

## 1 Introduction

Atrial fibrillation is the most common cardiac arrhythmia in adults (Chugh et al. 2014) and is associated with increased mortality and morbidity. Higher mortality is linked to associated heart failure and cardioembolism (Kornej et al. 2021).

The treatment of AF depends on the AF type (Calkins et al. 2012; January et al. 2014), presence of left ventricular dysfunction, patient symptoms, and preference. In patients with nonparoxysmal AF, one of the initial treatment strategies that has been and is still widely used is electrical cardioversion (ECV), which is fast and easy to perform.

The acute effect of ECV is excellent, and sinus rhythm is restored in more than 90% of patients. However, its mid‐term and long‐term efficacy is limited and the recurrence rate is high, for example, AF recurs in up to 50% of patients during the first 3 months, and most recurrences occurred within weeks of the procedure (Purkayastha et al. 2023; Vikman et al. 2003).

Despite adequate anticoagulation, ECV is associated with a 0.3%–0.5% risk of stroke. While infrequent, anesthesia is not without potential complications. Therefore, ECV risk, while low, is not negligible. In patients with a very low chance for benefit, that is, with sinus rhythm maintenance in the short term, the performance of a procedure associated with potential risk is futile. Furthermore, needless ECV increases healthcare costs without positive benefits for the patient (Noseworthy et al. 2015).

Several clinical parameters are associated with a high risk for AF recurrence, such as age, AF duration, anti‐arrhythmic medication, and enlarged left or right atrium (Doring et al. 2023). However, all models tested based on clinical parameters have had limited predictive value. Currently, there is no clear, strong, and established tool to predict AF recurrence after ECV. In the past, several ECG parameters obtained from spectral analysis of fibrillatory atrial waves were tested and found to have moderate predictive value (Alcaraz et al. 2011). Surprisingly, there is limited data on the usefulness of vector‐cardiography (VCG) for predicting AF recurrence in AF patients. VCG provides very sensitive, three‐dimensional information regarding the electrical forces acting on the atria and ventricles.

The aim of the study was to test predictors of SR maintenance after ECV, using (1) clinical variables, (2) parameters obtained from spectral analysis of ECG fibrilatory waves before cardioversion, and (3) parameters obtained from VCG analysis of ECG tracings before ECV.

## 2 Methods

### 2.1 Study Design and Patients

The study was a prospective, single‐center cohort study. It was approved by the Institutional Review Board, and patients signed informed consent.

All consecutive elective patients with nonparoxysmal atrial AF (NPAF) referred for elective ECV were enrolled. Enrollment was from January 2022 to June 2023 (18 months).

Exclusion criteria were sinus rhythm on admission, unwillingness to participate, atrial flutter or other regular atrial tachycardia other than AF, and hemodynamic instability. Furthermore, we analyzed only elective hemodynamically stable patients; hemodynamically unstable patients admitted for emergent ECV were not included. The use of anti‐arrhythmic medication before the ECV was left to the discretion of the treating outpatient cardiologist.

A detailed medical history, laboratory, and echocardiography parameters were obtained before the procedure.

ECGs were recorded at rest (for at least 10 min) in a supine position before the ECV, and before any analgosedation or anesthesia was given. Recordings used standard ECGs with digital recording facility (EKG 200 Cardioline). Each recording was 10 s long and sampled at 1000 Hz.

The digital ECG recordings were subjected to spectral and VCG analysis.

### 2.2 Electrical Cardioversion

ECVs were performed in short general anesthesia without orotracheal intubation in the intensive care unit with continuous monitoring. The anesthesia consisted of IV propofol, hypnomidate, or thiopental. ECVs consisted of a QRS‐synchronized biphasic direct current shock with patches in anterolateral position starting with 200 J and increasing to 270 J (TEC 5631 Nihon Kohden, India) until sinus rhythm was achieved. If three ECV attempts did not lead to SR restoration, further DC applications were abandoned; however, the patients were not excluded from our analysis. All patients were monitored in the intensive care unit for at least 3 h after ECV and then discharged. The anti‐arrhythmic medication at discharge was left to the discretion of the treating physician.

### 2.3 ECG Holter Recording

All patients underwent 24‐h Holter monitoring 3 months after cardioversion, and an outpatient visit was used to assess SR maintenance. Arrhythmia recurrence during the follow‐up was defined as episodes of AF or atrial tachycardia lasting > 30 s either on the 3‐month Holter or during any other clinical assessment during the first 3 months; however, only documented AF episodes were considered as AF recurrence.

### 2.4 Spectral Analysis

AF activity was analyzed using spectral and wavelet analysis. The spectral features (denoted in general as Lead_PreProcessingMethod_Parameter, for example, V1_WT_DF or aVR_SP_nPeaks) were computed from signals from leads II, aVR, and V1.

In the case of spectral analysis, QRST complexes were automatically identified and delineated using an algorithm described by Martinez et al. (Martinez et al. 2004). The results of this automated delineation were subsequently reviewed and corrected by expert visual inspection. Identified QRST segments were then replaced by zeros before spectral computation. This zero‐replacement method avoids contamination of the atrial activity spectrum by ventricular components. We chose this approach over alternatives such as template matching or adaptive filtering due to its simplicity and robustness in suppressing residual QRS energy in the atrial frequency band (3–15 Hz), which is critical for accurate dominant frequency and organization measurements. The remaining signal segments were mean‐centered, and the amplitude spectrum between 3 and 15 Hz was computed (method abbreviation: SP). For wavelet analysis, the Maximal overlap discrete wavelet transform was computed from the entire signal with transform level 7 and subsequent multiresolution analysis from which the sum of the last two bands was used for further computations (method abbreviation: WT). The computed parameters included dominant frequency (DF, frequency of the highest spectral peak), organizational index (OI, the ratio of energy of the dominant frequency plus its multiples and total energy in the 3–15 Hz band), regularity index (RI, the ratio of energy of the dominant frequency and total energy in the 3 Hz to 15 Hz band), and the quality factor of the dominant spectral peak measured 2 dB below the maximum (Q2). Specifically, five features were used in the models: (1) V1_WT_DF, (2) V1_WT_Q2, (3) V2_WT_RI, (4) aVR_WT_OI, and (5) II_SP_DF.

### 2.5 Vectorcardiography

Using vectorcardiography, we extracted three features (dXMean, dYMean, and dZMean) from an average beat in vectorcardiographic leads. These features represent average signal slope (i.e., average derivative signal) in automatically optimized windows, and these windows differ per X, Y, and Z axis (Plesinger et al. 2025). These features describe the signal slope before a QRS complex (dXMean), during the QRS (dYMean), and between the QRS and T‐wave (dZMean).

The automatically optimized windows were defined individually for each axis (X, Y, and Z) based on the maximal slope detection in characteristic parts of the ECG cycle:

dXmean: computed from the interval preceding the QRS onset (P‐wave to QRS transition), dYmean: computed from the QRS complex duration, dZmean: computed from the interval after the QRS until the T‐wave onset.

The signal was first band‐pass filtered (0.5–40 Hz), and differentiation was performed using a central difference method. Window borders were automatically adapted based on signal morphology to optimize the detection of maximal slope segments.

### 2.6 Statistical Analysis

Continuous variables are expressed as the mean ± SD. Data sets with a normal (Gaussian) distribution were analyzed using the Student's *t*‐test, and those with a non‐Gaussian distribution were analyzed using the Mann–Whitney *U* test. Categorical variables are shown using absolute and relative frequencies and were compared using χ^2^ or Fisher's exact test, as appropriate. The significance level was defined as *p* < 0.05.

Univariate analysis was performed using the whole dataset; however, we split the data for modeling tasks. We identified two cases of missing values, and therefore, a reduced dataset with 78 cases was used. We used data split into a 60%–40% ratio for training and testing purposes; the data were standardized to bring them into a similar scale. The training part was used for feature selection by LASSO Logistic Regression (LLR), for training predictive LLR models, and optimal model selection. The optimal model was selected based on the Akaike Information Criterion (AIC) and AUC performance on the training set. The testing part was used only to evaluate the AUC performance of the resultant models.

## 3 Results

A total of 80 patients were enrolled, aged 70.2 ± 10.6 years, 49 (61%) men, with a CHA_2_DS_2_‐VASc score of 3.15 ± 1.6 and BMI of 29.7 kg/m^2^. Baseline clinical characteristics and comparison of groups are shown in Table 1. At the 3‐month follow‐up, AF recurrence was found in 36 (45%) patients. Patients with AF recurrence, compared to patients with SR maintenance, were older, had higher CHA_2_DS_2_‐VASc scores, larger LA size, and often had a history of uncontrolled arterial hypertension (for details, see Table 1).

**TABLE 1:** Baseline characteristics.

	Total cohort (*n* = 80)	SR maintenance (*n* = 44)	AF recurrence (*n* = 36)	*p*
Age (years)	70.2 ± 10.6	68.6 ± 11.5	70.1 ± 10.4	0.560
Female gender (%)	31 (38.8)	19 (44.2)	12 (32.4)	0.390
AF duration (months)	20.6 ± 16.2	19.9 ± 13.0	21.5 ± 19.4	0.660
BMI (kg/m^2^)	29.7 ± 5.4	29.5 ± 6.1	29.9 ± 4.7	0.700
Number of ECV	1.6 ± 0.9	1.7 ± 0.1	1.5 ± 0.7	0.320
CHA_2_DS_2_‐VASc score	3.15 ± 1.6	2.9 ± 1.4	3.4 ± 1.7	0.210
LA diameter (mm)	44.7 ± 6.3	44.4 ± 6.4	45.0 ± 6.2	0.553
Diabetes Mellitus (%)	23 (28.8)	14 (32.5)	9 (24.3)	0.570
Arterial hypertension (%)	59 (73.8)	33 (76.7)	26 (70.3)	0.690
LVEF (%)	55.6 ± 9.3	55.3 ± 10.5	55.9 ± 7.6	0.780
Thyreopathy (%)	13 (16.3)	6 (13.9)	7 (18.9)	0.760
History of MI	9 (11.3)	6 (13.9)	3 (8.1)	0.490

*Note:* To compare means in continuous variables, *t*‐test was used. To compare the distribution of cathegorical variables, chi‐square or the Fisher's exact test was used.

Abbreviations: AF = atrial fibrillation, BMI = body mass index, ECV = electrical cardioversion, LA = left atrium, LVEF = left ventricular ejection fraction, Mi = myocardial infarction.

### 3.1 Vector Cardiography and Spectral Analysis

In univariate analysis, dXmean (OR 2.84, 95% CI 1.04–8.03, *p* = 0.002), dYmean (OR 0.23, 95% CI 0.08–0.63, *p* < 0.001), and dZMean (OR 0.18, 95% CI 0.06–0.51, *p* < 0.001) were reliable predictors of SR maintenance. Feature selection via LLR resulted in a combination of dYmean and dZmean, leading to a model with an AUC value of 0.78 (test set). The list of VCG parameters used is shown in Table 2.

**TABLE 2:** VCG and Spectral analysis features.

VCG features	OR (95% CI)	*p*
dXmean	2.84 (1.04–8.03)	0.002
dYmean	0.23 (0.08–0.63)	< 0.001
dZMean	0.18 (0.06–0.51)	< 0.001
**f‐wave analysis features**	**OR (95% CI)**	***p***
V1_WT_D	0.21 (0.07–0.58)	< 0.001
V1_WT_Q2	0.28 (0.10–0.78)	< 0.001
V2_WT_RI	2.84 (1.04–8.03)	0.020
aVR_WT_OI	3.54 (1.28–10.25)	0.006
II_SP_DF	3.54 (1.28–10.25)	0.006

Abbreviations: aVR_WT_OI = Lead aVR_WT_organizational index; dXmean = describes signal before a QRS complex; dYmean = describes signal during the QRS; dZMean = describes signal between the QRS and T‐wave; II_SP_DF = Lead II_SP_dominant frequency; V1_WT_DF = Lead V1_WT_dominant frequency; V1_WT_Q2 = Lead V1_WT_quality factor of the dominant spectral peak measured 2dB below the maximum; V2_WT_RI = Lead V2_WT_regularity index.

Univariately, spectral analysis features showed the II_SP_DF as the best SR predictor (OR 3.54, 95% CI 1.28–10.25, *p* = 0.006). The list of spectral analysis features tested in univariate analysis is shown in Table 2. Spectral features led to a model combining II_SP_DF and V1_WT_Q2 in a model with AUC = 0.76 (test set).

### 3.2 Clinical Variables

Clinical variables, such as diabetes, obesity, arterial hypertension, and thyropathy, were evaluated univariately. Parameters obtained from echocardiography examinations, such as left ventricular ejection fraction, left atrial size, and valvular disease, were also included in the feature selection before LLR modeling. Univariately, neither of the clinical variables reached statistical significance. Furthermore, neither of these variables surpassed the feature selection, so an LLR model based only on clinical variables was not trained.

### 3.3 Combination of Spectral Analysis, VCG, and Clinical Parameters

**FIGURE 1:**
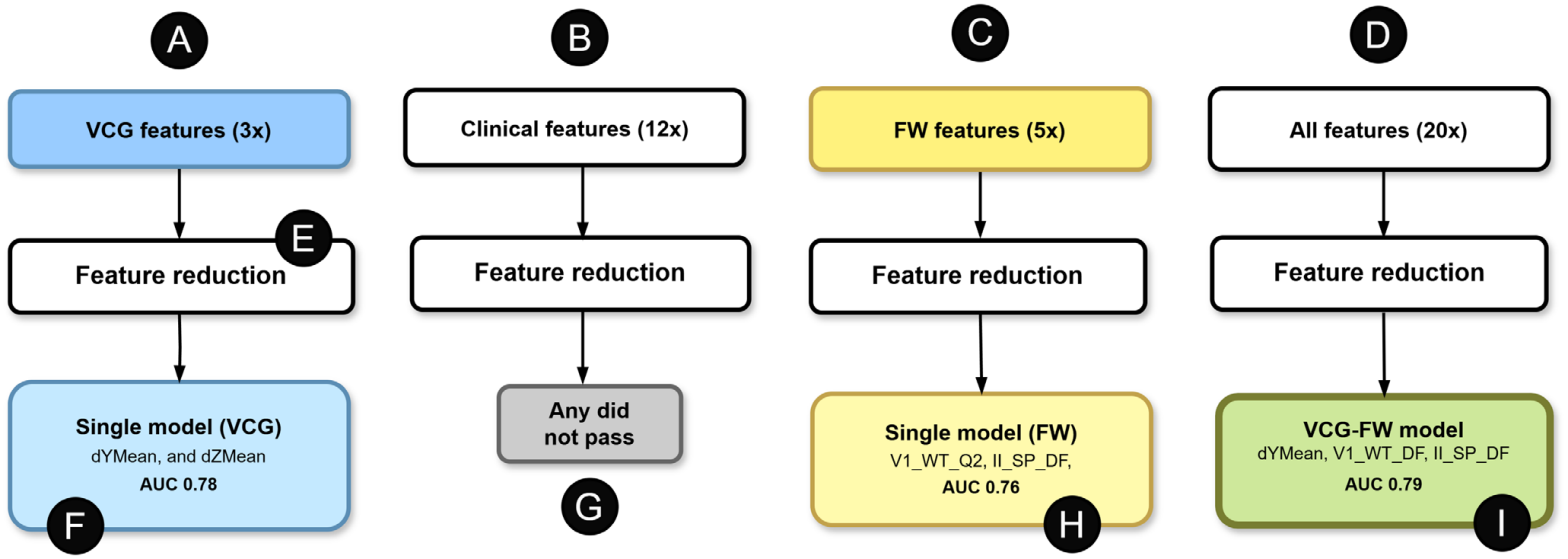
Features from different sources were used to build logistic regression models. Vectorcardiographic features (A) underwent feature reduction (E) and formed the VCG model (F); fibrillatory waves‐based features (C) formed the FW model (H). The joint use of all features (D), led to the combined model with improved performance (I). Clinical features (B) did not pass (G).

**FIGURE 2:**
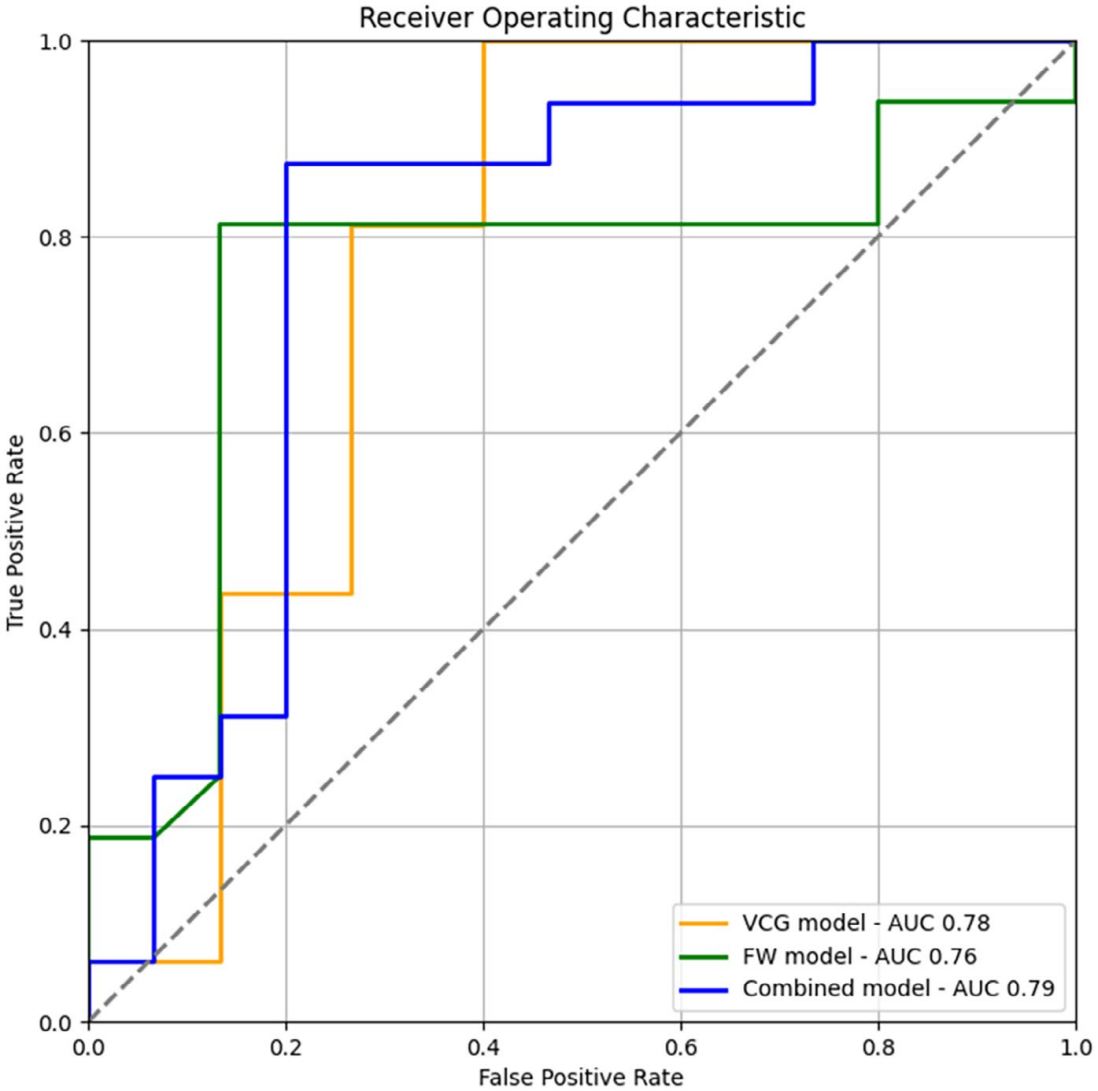
AUC (area under curve) values graph.

Feature selection from all variables (including clinical) resulted in three features: dYmean, II_SP_DF, and V1_WT_DF. These variables were then used to construct a final LLR model, which achieved an AUC of 0.79 on the test set (see Figures 1 and 2).

## 4 Discussion

Our study showed that a combination of spectral analysis and VCG parameters offers more predictive power regarding AF recurrence after cardioversion than spectral analysis, VCG, or clinical parameters alone.

Atrial fibrillation remains the most common arrhythmia in clinical practice, with all treatment modalities having only moderate effect. Noninvasive risk stratification of AF patients is usually based only on clinical characteristics, and with regard to ECG‐based predictors, most of them have been studied during sinus rhythm.

### 4.1 Spectral Analysis Parameters

Shortening of atrial refractoriness is considered a hallmark of electrical remodeling during AF. Although it was not validated whether the effective refractory period during SR equals that during AF, many excitations occurring during the repolarization phase are considered to shorten atrial refractoriness, and as such, the average atrial fibrillatory rate is likely to reflect the average refractoriness. Spectral analysis offers an analysis of atrial fibrillation. After filtering and QRS subtraction, several features, such as dominant frequency (DF), regularity index (RI), and the organizational index (OI), are measured.

DF, defined as the peak with the highest amplitude in the power spectrum (Berenfeld 2007), has been repeatedly confirmed as a good predictor for SR maintenance. Lower DF corresponds to more organized signals. Not surprisingly, DF is lower in patients with paroxysmal AF compared to nonparoxysmal AF (Sanders et al. 2005; Xi et al. 2004).

Higher DF has been associated with a higher risk of AF recurrence after cardioversion or catheter ablation, which is in agreement with our results (Yoshida et al. 2011).

While most previous reports emphasize the relevance of DF after catheter ablation, some studies have directly evaluated DF's role following ECV. Langberg et al. demonstrated (using spectral analysis of surface ECG) that dominant frequency and signal organization were predictive of AF recurrence following cardioversion (Langberg et al. 1998). While DF does not directly measure refractoriness, it appears to reflect atrial electrophysiological properties associated with AF organization and maintenance, which are key determinants of sinus rhythm stability after ECV. Furthermore, Tieleman et al. linked early AF recurrences post‐ECV to atrial electrical remodeling and shortening of atrial refractoriness induced by AF itself, suggesting that atrial vulnerability plays a crucial role in post‐cardioversion recurrence (Tieleman et al. 1998). Taken together, while DF does not directly measure atrial refractoriness, these studies indicate that it reflects underlying atrial electrophysiological remodeling and may serve as a practical surrogate marker for predicting AF recurrence after ECV.

Lankveld et al. (Lankveld et al. 2016) found that lower DF and higher OI were significant predictors of AF termination during catheter ablation. Alcaraz et al. (Alcaraz et al. 2016) showed that more organized AF, defined by higher OI, also predicted long‐term maintenance of sinus rhythm after catheter ablation. According to a report by the same group, a combination of f‐wave amplitude during AF along with DF was the best ECG predictor of SR maintenance after cardioversion for AF (Alcaraz and Rieta 2009). Our study is in agreement with all aforementioned studies showing that spectral parameters can predict SR maintenance after cardioversion.

### 4.2 VCG Parameters

Vectorcardiography is a sensitive method for analyzing three‐dimensional information associated with the electrical forces in the ventricles. It provides a valuable and very sensitive description of ventricular activation, which can detect the preclinical damage in the ventricles.

However, associating ventricular depolarization acquired by VCG with AF recurrence is not intuitive; this may be the reason why, to the best of our knowledge, only our previous study (Plesinger et al. 2025) reported VCG parameters to be significant in SR restoration.

In this study, we followed our previous approach to extract three VCG features (dXMean, dYMean, and dZMean). They describe the averaged derived signal in automatically optimized windows. dXMean describes the signal before a QRS complex, dYMean during the QRS, and dZMean between the QRS and T‐wave. However, the selected VCG model was combined only from dYMean and dZMean (and a bias term), suggesting that QRS morphology, together with T‐wave onset, is associated with early recurrence of AF after ECV.

dYmean reflects signal changes during the QRS complex (ventricular depolarization), and dZmean reflects changes between QRS and T‐wave (early repolarization phase). Lower dYmean and dZmean may indicate greater ventricular electrical heterogeneity or subtle conduction abnormalities, which could promote atrial remodeling or impaired atrioventricular coupling, favoring AF recurrence. Conversely, higher dYmean and dZmean values could reflect more synchronized, homogeneous ventricular activation and recovery, thus supporting more stable sinus rhythm after ECV.

As was shown previously, both the atrium‐related and ventricle‐related ECG variables present risk factors for AF recurrences. Among standard ECG parameters, for example, ventricular hypertrophy, QRS notching, intraventricular conduction delay, or ST‐T abnormalities, were found to be risk factors of incident AF (Aizawa et al. 2017).

Although VCG primarily reflects ventricular depolarization and repolarization patterns, increasing evidence suggests that abnormalities in ventricular electrical activity may indirectly indicate atrial vulnerability. Several studies support this concept. For instance, Shah et al. and Li et al. identified left ventricular hypertrophy as an independent predictor of AF recurrence. Fragmented QRS complexes, as shown by Eren et al., also predict recurrence, suggesting a role for ventricular conduction abnormalities. Our previous work (Plesinger et al.) demonstrated that VCG‐derived slopes during the QRS‐T segment can predict AF recurrence, likely reflecting subclinical ventricular remodeling, which may influence atrial electrophysiological stability.

Additional support comes from MRI‐based studies (e.g., Suksaranjit et al.) showing that ventricular fibrosis predicts AF recurrence. Factors unrelated to direct atrial function, such as arterial hypertension, have consistently been identified as potent predictors of AF recurrence in numerous reports. This reinforces the notion that AF is a multifactorial disease influenced by both atrial and ventricular factors. VCG can sensitively detect such changes, making it a valuable noninvasive marker of arrhythmogenic risk. In summary, although VCG does not directly assess atrial function, it reflects ventricular pathology that may contribute to atrial remodeling and AF recurrence.

### 4.3 Clinical Characteristics in Prediction of AF Recurrences

Several clinical predictors of AF recurrence after ECV have been previously identified, for example, higher age, AF duration, and left atrial size. According to the meta‐analysis with 2725 patients after ECV, the left atrial volume index (LAVI) was the most significant predictor of AF recurrence. Each unit increase in LAVI resulted in an increase in the risk of AF recurrence by 6% (Raniga et al. 2024).

In our patients, neither age nor LA dimensions was a predictor of SR maintenance after ECV. In contrast to the strong predictive values of LAVI as an independent predictor of AF recurrence, which was confirmed in many previous studies (Toufan et al. 2017), the findings on LA are not so consistent. While some reports demonstrated LA as a predictor of AF recurrences, others did not confirm these findings. For example, in the report by Lin et al., LA size did not predict AF recurrences after cardioversion (Lin et al. 2002). Park et al. studied morphological (LA size, LAVI) and functional (electromechanical conduction time) LA parameters in the prediction of AF recurrences after electrical cardioversion. Both types of LA parameters were associated with a higher risk of AF recurrence, but in the multivariate model, only electromechanical conduction time remained an independent predictor (Park et al. 2010). In our study, the absence of predictive value of LA size (and even more probably of age) could have been caused by a small sample size, and unfortunately, LAVI was not determined in all patients. Although the sample size of this study was limited, it supports the importance of electrical parameters over morphological changes.

In a univariate analysis, none of the clinical parameters was able to separate ECV responders (i.e., patients with early AF recurrence) and nonresponders. Furthermore, feature selection by LASSO did not find any suitable set of clinical features that could form a reliable model, and as such, a model trained purely on clinical data was not trained.

### 4.4 Combination of ECG and Clinical Parameters

As noted, when using a multivariate LLR model, clinical parameters combined with ECG characteristics were not selected as predictors of SR maintenance, while spectral and VCG features had substantial power to predict SR maintenance.

The predictive power of the combination of spectral analysis with VCG regarding AF recurrence can be explained by the comprehensiveness of information obtained from these two techniques. Spectral analysis probably offers the best assessment of the degree of atrial impairment, while VCG offers a detailed analysis of ventricular impairment. The reason ventricular impairment plays a significant role in AF recurrence is complex. However, as shown in previous AF studies, left ventricular hypertrophy or ST‐T segment abnormalities are associated with a higher risk for AF (Berry‐Noronha et al. 2024). As such, both ECG analysis techniques can independently predict AF recurrence; however, the predictive power and AUC are improved when the techniques are combined.

Regarding clinical use and implications, several Holter devices are spectral analysis capable. If our data were confirmed in a larger sample size, then the use of more sophisticated Holter analysis could identify patients at very high or very low risk for AF recurrence and could significantly improve clinical decision‐making.

Recently, an AI‐based model to predict AF has been developed. For example, an AI‐enabled ECG acquired during sinus rhythm permits identification at the point of care of individuals with AF. (Attia et al. 2019). However, AI‐based models typically require very large datasets and complex computational infrastructure. In contrast, our approach using spectral and VCG analysis offers a clinically practical and explainable alternative that can be implemented with conventional ECG technology.

## 5 Study Limitations

Consecutive patients admitted for electrical cardioversion were studied; therefore, our study cohort represents a mixed population (including patients with previous catheter ablation). On the other hand, it reflects the real population currently treated with electrical cardioversion.

Indexed left atrial volume (LAVi) was not available in all patients and, therefore, not used. Only LA dimensions were put in our logistic model. Our sample size was limited, and our results should be confirmed in a larger sample of AF patients.

## 6 Conclusion

Comprehensive digital analysis of ECG and VCG waveforms significantly enhances the predictive performance of models compared to those based solely on conventional clinical and echocardiographic parameters. Additionally, integrating spectral analysis of fibrillatory waves with vectorcardiographic characterization of QRS complexes offers superior predictive capability over univariate or single‐modality approaches.

## Author Contributions

S.H. conceptualized the study, coordinated the project, and wrote the manuscript. F.P. developed and performed the vectorcardiographic analysis. Z.B. and J.V. conducted the spectral analysis. M.H. and D.H. contributed to patient recruitment and data collection. V.F. assisted with data preprocessing and quality control. P.O. supervised the project, contributed to study design and interpretation, and critically revised the manuscript. All authors reviewed and approved the final version of the manuscript.

## Ethics Statement

The study was approved by the local Ethics Committee. Each patient signed informed consent.

## Conflicts of Interest

The authors declare no conflicts of interest.

## Data Availability

The data that support the findings of this study are available from the corresponding author upon reasonable request.
